# Outcomes of 3,400 patients with cancer admitted to intensive care unit: a Brazilian prospective study

**DOI:** 10.1186/cc10153

**Published:** 2011-06-22

**Authors:** LA Hajjar, F Galas, J Almeida, D Nagaoka, FA Duarte, RE Nakamura, C Simoes, R Kalil-Filho, PM Hoff, JOC Auler

**Affiliations:** 1 ICESP, São Paulo - SP, Brazil

## Background

Intensive care unit (ICU) admission of critically ill cancer patients was controversial until recently. In the last years, advances in the management of malignancies and organ failures have improved outcomes of patients, resulting in higher rates of survival in the ICU. The aim of this study is to prospectively evaluate the characteristics, short and midterm outcomes of cancer patients requiring intensive care.

## Methods

During 2 years, we evaluated prospectively patients with cancer admitted to the Instituto do Cancer do Estado de São Paulo. A total of 3,400 patients were included in the study; and collected data were baseline data, risk scores, clinical status, co-morbidities, admission diagnosis, ICU interventions, ICU and hospital outcomes and 90-day outcomes.

## Results

From 3,400 patients, 52.8% had solid tumors and 47.2% had hematologic malignancies. The most frequent reasons for ICU admission were: sepsis (32%), postoperative care (27%) and respiratory failure (21%). The mean APACHE II score value 24 hours after admission was 23.1 ± 7.8 (8 to 45). ICU mortality was 22%, hospital mortality was 31% and 3-month mortality was 44%. Logistic regression analysis showed that need for mechanical ventilation (odds ratio = 7.76; 95% CI = 4.56 to 12.85), presence of metastasis (odds ratio = 2.87; 95% CI = 2.06 to 5.28), occurrence of acute renal failure (odds ratio = 2.92; 95% CI = 1.67 to 9.46) and higher SOFA scores 72 hours after admission (odds ratio = 6.76; 95% CI = 5.56 to 13.85) were independently associated with increased hospital mortality. The 3-month quality of life of patients who survived was considered unchanged in 51% patients, worse in 25% and better in 24%.

## Conclusion

This prospective analysis of 3,400 patients with cancer needing intensive care shows high survival rates and good quality of life after ICU admission. These data encourage intensive care treatment in oncologic patients to prevent, detect and cure organ dysfunction.

